# NEW BEREAVEMENT IS A RISK FACTOR FOR BINGE DRINKING, SMOKING, AND POOR MENTAL HEALTH (2019 GEORGIA BRFSS)

**DOI:** 10.1093/geroni/igac059.1371

**Published:** 2022-12-20

**Authors:** Toni Miles

**Affiliations:** Rosalynn Carter Institute, Athens, Georgia, United States

## Abstract

This presentation advances studies of population health by estimating the association between new bereavement and binge drinking rates among adults aged 50 years and older. In other reports, bereavement is associated with significantly higher rates of binge drinking among older adults. In 2019, the state of Georgia used an optional module in the Behavioral Risk Factor Surveillance Survey (BRFSS) to measure the prevalence of new bereavement. This is the first effort to capture population-level data on bereavement in Georgia. 45% of adults (4 million persons) aged 18 and older were newly bereaved in the 24 months prior to survey. Highest bereavement rates: adults aged 55 to 64 years (51%), unemployed (49%), and Black respondents (60%). Binge drinking rate for bereaved was 31 % (versus 23.6% not bereaved). Combined bereavement and binge drinking increased risk for poor mental health (OR = 2.83) and smoking (OR = 4.54).

